# Relation of Aortic Waveforms with Gut Hormones following Continuous and Interval Exercise among Older Adults with Prediabetes

**DOI:** 10.3390/metabo13020137

**Published:** 2023-01-17

**Authors:** Daniel J. Battillo, Steven K. Malin

**Affiliations:** 1 Department of Kinesiology and Health, Rutgers University, New Brunswick, NJ 08901, USA; 2 Department of Kinesiology, University of Virginia, Charlottesville, VA 22903, USA; 3 Division of Endocrinology, Metabolism & Nutrition, Rutgers University, New Brunswick, NJ 08901, USA; 4 New Jersey Institute for Food, Nutrition and Health, Rutgers University, New Brunswick, NJ 08901, USA; 5 Institute of Translational Medicine and Science, Rutgers University, New Brunswick, NJ 08901, USA

**Keywords:** obesity, type 2 diabetes, arterial stiffness, endothelial function, physical activity

## Abstract

Prediabetes raises cardiovascular disease risk, in part through elevated aortic waveforms. While insulin is a vasodilatory hormone, the gut hormone relation to aortic waveforms is less clear. We hypothesized that exercise, independent of intensity, would favor aortic waveforms in relation to gut hormones. Older adults (61.3 ± 1.5 yr; 33.2 ± 1.1 kg/m^2^) with prediabetes (ADA criteria) were randomized to undertake 60 min of work-matched continuous (CONT, *n* = 14) or interval (INT, *n* = 14) exercise for 2 wks. During a 180 min 75-g OGTT, a number of aortic waveforms (applanation tonometry) were assessed: the augmentation pressure (AP) and index (AIx75), brachial (bBP) and central blood pressure (cBP), pulse pressure (bPP and cPP), pulse pressure amplification (PPA), and forward (Pf) and backward pressure (Pb) waveforms. Acylated-ghrelin (AG), des-acylated ghrelin (dAG), GIP, and GLP-1_active_ were measured, and correlations were co-varied for insulin. Independent of intensity, exercise increased VO_2_peak (*p* = 0.01) and PPA_120min_ (*p* = 0.01) and reduced weight (*p* < 0.01), as well as AP_120min_ (*p* = 0.02) and AIx75_120min_ (*p* < 0.01). CONT lowered bSBP (*p* < 0.02) and bDBP (*p* < 0.02) tAUC_180min_ more than INT. There were decreases dAG_0min_ related to Pb_120min_ (r = 0.47, *p* = 0.03), cPP_120min_ (r = 0.48, *p* = 0.02), and AP_120min_ (r = 0.46, *p* = 0.02). Declines in AG tAUC_60min_ correlated with lower Pb_120min_ (r = 0.47, *p* = 0.03) and cPP_120min_ (r = 0.49, *p* = 0.02) were also found. GLP-1_active 0min_ was reduced associated with lowered AP_180min_ (r = 0.49, *p* = 0.02). Thus, while CONT exercise favored blood pressure, both intensities of exercise improved aortic waveforms in relation to gut hormones after controlling for insulin.

## 1. Introduction

Aortic waveforms are clinically relevant as they can reflect the load on the heart and/or compliance of peripheral vessels [1,2,3,4,5]. While pulse-wave velocity is considered the gold standard for arterial stiffness, the augmentation index (AIx75) is a surrogate measure that enables pulse-wave reflection for the understanding of central and peripheral hemodynamics [6]. AIx75 is thus influenced by central hemodynamics coupled with the peripheral arterial tree. Pressure waves in the aorta can be separated into forward (Pf) and backward waves (Pb). Pf is generated mainly by left ventricular contraction and pulse-wave velocity, while backward pressure (Pb) is caused by the reflection of the Pf back toward the heart due to varying characteristics of the vascular walls [4,5]. This is clinically relevant towards understanding pulsatile components that influence central (aortic) and peripheral (brachial) blood pressure, leading to CVD risk among older adults [7]. To date, though, most research on aortic waveform components has focused on fasting measures and have not considered the post-prandial state. This is physiologically important as the post-prandial state is considered a stronger predictor of cardiovascular disease (CVD) than the fasting state alone [8]. 

Insulin is the prevailing post-prandial hormone secreted from pancreatic beta-cells in response to carbohydrate and protein. It plays a key role in maintaining blood glucose levels and promoting arterial compliance of blood vessels [9]. Arterial compliance is an important mechanical property that contributes to regulation of blood pressure, flow, and hemodynamic load on the heart [4,10]. Despite insulin acutely lowering AIx75 and pulse wave velocity in healthy individuals [10], there is reduced endothelial responsiveness in some [11,12], but not all [13,14,15], studies of adults with obesity. In turn, this has raised questions on the possibility that other post-prandial hormones could influence vascular function. Ghrelin is often recognized as an appetite-stimulating hormone secreted from oxyntic glands in the stomach. Interestingly, exogenous ghrelin exerts beneficial hemodynamic effects in healthy participants [16], as well as those with congestive heart failure, [17] through, in part, the inhibition of proinflammatory cytokines [18]. In fact, ghrelin administration increased vasodilation in response to acetylcholine via a nitric oxide specific mechanism [19] in people with metabolic syndrome. Glucagon-like peptide (GLP-1), an established incretin with known effects to promote beta-cell insulin secretion and delayed gastric emptying, also increases macro- and micro-vascular dilatory effects with and without insulin [20]. Additionally, glucose-dependent insulinotropic polypeptide (GIP), a small-intestinal K cell derived hormone known to also increase insulin secretion, is noted to raise adipose tissue blood flow during conditions of hyperglycemia and hyperinsulinemia, although these effects may be attenuated in people with obesity [21,22,23].

Aerobic exercise improves blood pressure (BP), lipid profiles, and inflammation, often in the absence of clinically meaningful weight loss [24,25,26]. Furthermore, acute exercise can decrease fasting AIx75 during the immediate post-exercise period [27], although some suggest the acute effect of aerobic exercise on AIx75 may last up to 24 h following the last bout [28]. Lower AIx75 following exercise may be partially attributed to the working muscles promoting reduced vascular resistance via enhancement of nitric oxide [29,30]. We also have shown that short-term interval (INT) exercise training reduces AIx75 during the post-prandial, but not fasting, state in people with obesity [31]. While our later work suggested that insulin may have, in part, contributed to these favorable reductions in post-prandial AIx75, independent of exercise intensity, it is unknown whether ghrelin and/or incretins play a role in aortic waveform changes following exercise, independent of insulin. Furthermore, we did not determine if changes in AIx75 were depicted by improved Pf or Pb waveforms, nor did we assess central compared with brachial blood pressures to discern pulse pressure amplification—a CVD mortality risk factor [32]. Thus, we tested herein the hypothesis that exercise, independent of intensity, would reduce post-prandial aortic waveforms in older adults with prediabetes. We further hypothesized this change would relate to changes in gut hormones implicated in regulating vascular function. 

## 2. Methods

### 2.1. Participants

Twenty-eight older adults with obesity (61.3 ± 1.5 yr; 33.2 ± 1.1 kg/m^2^, Table 1) were recruited via advertisements. Some of the AIx75 related outcomes, gut hormones, and cardiometabolic data were previously reported [31,33,34]. Participants were screened for prediabetes based on the American Diabetes Association criteria (75g OGTT) and had to have impaired fasting glucose (100–125 mg/dL), impaired glucose tolerance (2-hr plasma glucose 140–200 mg/dL), and/or elevated HbA1c (5.7–6.4%). Participants were non-smoking, sedentary (exercise < 60 min/wk), and weight stable over the prior six months (≤2 kg variation). People were excluded if they had chronic disease (i.e., renal, hepatic, cardiovascular, etc.) or were on anti-diabetic or weight-inducing medications (e.g., GLP-1 agonists, sulfonylureas, biguanides, etc.). All participants underwent a physical exam and stress test with an electrocardiogram to ensure their health status. Individuals provided written and verbal informed consent before participation as approved by the University of Virginia Institutional Review Board (IRB-HSR #17822).

### 2.2. Aerobic Fitness and Body Mass

Peak oxygen consumption (VO_2_peak) and heart rate (HRpeak) were determined using a continuous incremental cycle ergometer exercise test and indirect calorimetry (Carefusion, Vmax Encore, Yorba Linda, CA, USA) as described previously [31,33,34]. Body weight was measured to the nearest 0.01 kg on a digital scale while height was measured with a stadiometer to assess body mass index (BMI). 

### 2.3. Metabolic Control

Participants were instructed to refrain from alcohol, caffeine, medication, and strenuous physical activity for 24 h prior to each study visit. Participants were also instructed to consume a diet containing approximately 250 g of carbohydrates during the 24 h period prior to the pre-intervention testing to minimize influence on alterations in insulin secretion and gut hormones. This diet was recorded and replicated on the day before post-intervention testing. Participants were instructed to maintain non-exercise physical activity and habitual diets throughout the intervention.

### 2.4. OGTT

Participants reported to the Clinical Research Unit (CRU) after an approximate 10 h overnight fast. An IV catheter was placed in the antecubital fossa for blood draws to determine glucose and hormonal responses during a 75 g oral glucose load. Blood was collected at 0, 30, and 60 min to capture acylated and des-ghrelin, GLP-1_active_, and GIP [32,33], while glucose and insulin were additionally recorded at 90, 120, and 180 min. Post-intervention assessments were obtained approximately 24 h after the last training session.

### 2.5. Pulse Waveform Analysis

The SphygmoCor XCEL system (AtCor Medical, Itasca, IL, USA) was used to characterize hemodynamic and aortic waveform responses, as described before [24]. In short, this included peripheral systolic (bSBP), diastolic (bDBP) and pulse pressure (bPP), heart rate (HR), central systolic (cSBP), diastolic (cDBP) and pulse pressure (cPP), and the augmentation index (AIx), as well as wave deconvolution aspects of forward (Pf) and backward (Pb) pressure and reflection magnitude (RM). The augmentation index was corrected to a standard HR of 75 bpm (AIx75) using the manufacture’s software. Pulse pressure amplification (PPA) was calculated as a ratio (brachial PP/central PP). All measurements occurred while individuals were resting quietly in a semi-supine position in a temperature-controlled room. A blood pressure cuff was placed on upper arm and measurements were recorded in triplicate over a 10 min period and averaged. tAUC for aortic waveform measures was calculated from the values obtained at 0, 60, 120, and 180 min of the OGTT. 

### 2.6. Exercise Training

Participants were randomly assigned to either supervised CONT or INT training, utilizing a block design that was stratified by a prediabetes phenotype. Twelve work-matched bouts of cycle ergometry exercise were performed for 60 min/d over thirteen days. CONT exercise was performed at an intensity of 70% HRpeak; whereas INT exercise involved alternating 3 min intervals at 90% HRpeak followed by 50% HRpeak for the 60 min duration. The first 2 exercise sessions, however, were performed at 30 and 45 min, respectively, at the desired intensity to ease participants into the intervention. Ad-libitum water, but no food, was provided to the subjects. Heart rate (Polar Electro, Inc. Woodbury, NY) and rating of perceived exertion (RPE) were monitored throughout exercise to ensure appropriate intensity. Energy expenditure during CONT and INT exercise was calculated using HR-VO_2_ regression analysis, as previously described [34]. 

### 2.7. Biochemical Analysis 

Plasma glucose was measured immediately after collection using the glucose oxidase method (YSI 2300 STAT Plus, Yellow Springs, OH, USA). Blood samples were collected in chilled vacutainers that contained protease inhibitors. AG and dAG samples contained aprotinin, DPP-IV, and AEBSF (EMD Millipore, Billerica, MA, USA). GLP-1 contained aprotinin and DPP-IV, while insulin contained only aprotinin. Blood was centrifuged at 4 °C for 10 min at 3000 RPM. Following centrifugation, HCl was immediately added to acidify the ghrelin sample. All blood was frozen at −80°C until subsequent analysis. AG and dAG concentrations, as well as GLP-1_active_ and insulin, were determined using an enzyme-linked immunosorbent assay (ELISA), as described before [32]. 

### 2.8. Statistical Analysis

Data were analyzed using GraphPad Prism version 9 (GraphPad Software, San Diego, CA, USA). Non-normally distributed data were log-transformed for analysis. Baseline differences were assessed using independent samples, two-tailed *t*-test, while repeated measures analysis of variances (ANOVA) was used to determine group x time differences. Pearson correlations were used to examine relationships, and insulin was used as a co-variate for gut hormones to isolate effects. Statistical significance was accepted as *p* ≤ 0.05 and data are presented as mean ± SEM. 

## 3. Results

### 3.1. Participant and Exercise Training Characteristics

Independent of intensity, exercise raised VO_2_peak (*p* < 0.01) and decreased BMI (*p* < 0.01; Table 1). Exercise session adherence was excellent and similar between CONT and INT (96.2 ± 2.2% vs. 95.6 ± 1.5%; *p* = 0.83). Despite INT having a higher heart rate during training compared with CONT (77.8 ± 1.0% vs. 72.6 ± 1.3%; *p* < 0.01), there were no significant differences between CONT and INT in RPE (12.5 ± 0.3 vs. 12.0 ± 0.5 a.u.; *p* = 0.46) or exercise energy expenditure (393.2 ± 16.3 vs. 384.5 ± 18.9 kcal/session; *p* = 0.62). 

### 3.2. Glucose Tolerance and Insulin

Although exercise did not reduce fasting glucose, it reduced both 120 min glucose (*p* = 0.02) and glucose tAUC_180min_ (*p* = 0.03), independent of intensity (Table 1). Furthermore, fasting insulin was not altered, but insulin tAUC_180min_ was significantly reduced following both exercise intensities (*p* < 0.01; Table 2). 

### 3.3. Hemodynamics

**Figure 1:**
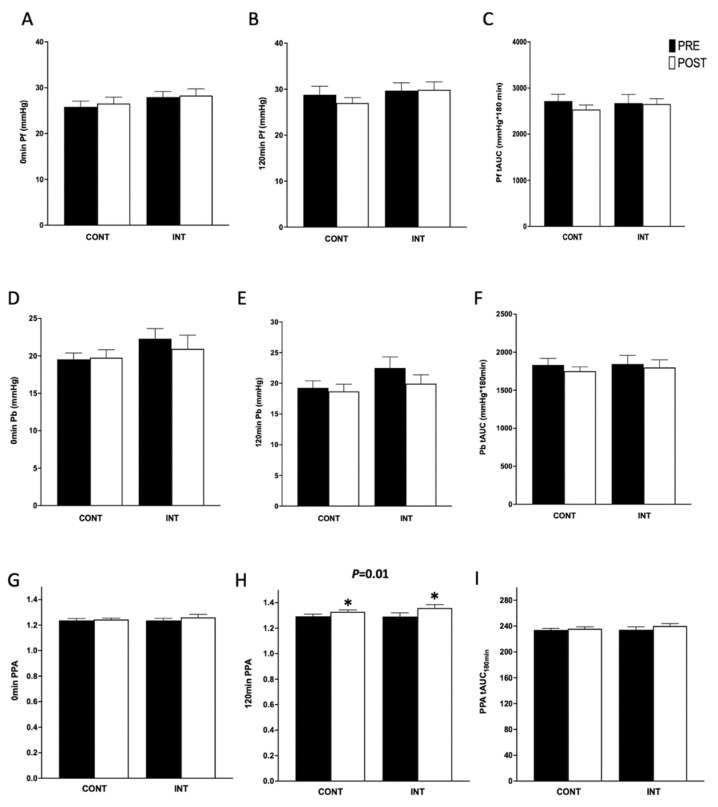
Effect of continuous (CONT) and interval (INT) exercise on measures of aortic waveforms and central hemodynamics. Exercise did not alter forward (**A**–**C**) or backward (**D**–**F**) pressure waveforms in the fasted or post-prandial states. While there were no changes to fasting (**G**) or tAUC (**I**) pulse pressure amplification (PPA), exercise, regardless of intensity, increased 120 min PPA (**H**). Data are mean ± SEM. * Denotes a significant (*p* ≤ 0.05) difference between pre- and post-exercise conditions.

We report that AIx75 tAUC_180min_ was lowered after both INT and CONT exercise (*p* < 0.01; Figure 1), independent of heart rate changes in response to exercise (*p* = 0.66 and *p* = 0.94, respectively; Table 3). CONT training elicited greater improvements than INT in both bSBP tAUC_180min_ (*p* = 0.02) and bDBP tAUC_180min_ (*p* = 0.04; Table 3). However, CONT and INT comparably reduced AP_120min_ (*p* = 0.02) and increased PPA_120min_ (*p* = 0.01), although there was no influence on 120 min bSBP (*p* = 0.57) and cSBP (*p* = 0.96), or 120 min bDBP (*p* = 0.45) and cDBP (*p* = 0.33; Table 3). 

### 3.4. Gut Hormones

Fasting GIP increased with CONT but decreased after INT (*p* = 0.03). However, there were no exercise-induced changes to GIP tAUC_60min_ (*p* = 0.93; Table 2). Furthermore, there was no significant effect of CONT or INT on fasting AG (*p* = 0.32) or dAG (*p* = 0.66), nor AG or dAG tAUC_60min_ after OGTT administration (*p* = 0.20 and *p* = 0.72, respectively). Additionally, neither exercise intervention altered fasting or tAUC_60min_ GLP-1_active_ (*p* = 0.38 and *p* = 0.73, respectively; Table 2). 

### 3.5. Correlations

**Figure 2:**
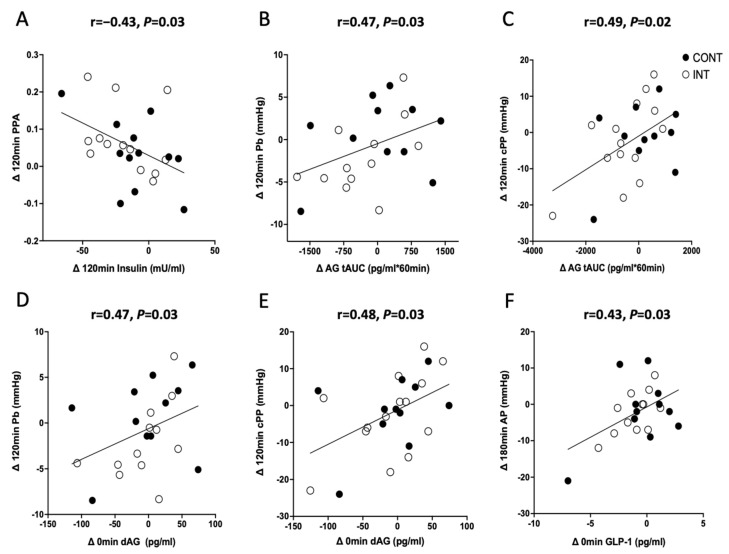
Correlations in fasted and post-prandial gut hormone changes following the intervention. The change (∆) in insulin_120min_ to the ∆ in pulse pressure amplification (PPA)_120min_ (**A**). The ∆ in acylated-ghrelin (AG) tAUC to the ∆ in backward pressure (Pb)_120min_ (**B**). The ∆ in AG tAUC to the ∆ in central pulse pressure (cPP)_120min_ (**C**)_._ The ∆ in fasting des-acylated ghrelin dAG to the ∆ in Pb_120min_ (**D**). The ∆ in fasting dAG to the ∆ in cPP_120min_ (**E**). The ∆ in fasting glucagon-like peptide (GLP-1) to the ∆ in augmentation pressure (AP)_180min_ (**F**).

Exercise-induced reductions in fasting insulin correlated with lower Pf_120min_ (r = 0.54, *p* = 0.01; Figure 2). Lower Pb_120min_ correlated with declines in dAG_0min_ (r = 0.47, *p* = 0.03) and AG tAUC_60min_ (r = 0.47, *p* = 0.03; Figure 2). Prior to covarying for insulin, however, reductions in Pb_120min_ correlated with neither dAG_0min_ (r = 0.37, *p* = 0.08) nor AG tAUC_60min_ (r = 0.30, *p* = 0.18). Furthermore, reduced GLP-1_active 0min_ was associated with lowered AP_180min_ after covarying for insulin (r = 0.49, *p* = 0.02; Figure 2) but not before (r = 0.32, *p* = 0.19), and increased GLP-1_active_ tAUC_60min_ was associated with decreased Pf_180min_ (r = −0.51, *p* = 0.03; Figure 2) but not before (r = −0.18, *p* = 0.43). 

## 4. Discussion

The primary finding from the present study is exercise, independent of intensity, reduced post-prandial AIx and AP, as well as increased post-prandial PPA in older adults with prediabetes. However, CONT exercise yielded lower blood pressure responses during the OGTT than INT. This contrasts prior work suggesting INT may be better at reducing fasted blood pressure, particularly during the immediate post-exercise period (~1 h) [35]. The exact cause of improved post-prandial blood pressure following CONT and INT is beyond the scope of this work, but we [36] and others have reported that CONT exercise favors increased conduit artery blood flow during an OGTT [37] and/or brachial flow-mediated dilation [38]. As flow-mediated dilation is a non-invasive measure of nitric oxide bioavailability, it is possible that the rhythmic nature of muscle contraction during CONT exercise promoted endothelial function. In either case, few data are available examining aortic waveforms following short-term exercise [39,40,41]. Our current findings of no change in Pf or Pb contrast some prior work displaying reduced Pf and Pb following lower body exercise in the immediate post-exercise period for up to 2 h [41]. However, this latter study was conducted in participants who were young, healthy adults (26.0 ± 3.0 yr), and aortic waveform measures were taken every 20 min up to 2 h after exercise, thereby making comparisons difficult to our older participants (61.3 ± 1.5 yr). Taken together with this immediate post-exercise work, our data suggests these effects are short-lived, potentially since we observed no effect on fasting indices 24 h after the last training session. Another study looking at resistance exercise in healthy adults on aortic waveforms reported that AIx increased 1 h following the bout [40], and other work saw similar increases in AIx 10 min after a bout of resistance exercise, but no changes in central or brachial blood pressures [39]. The mechanisms mediating this contrary response to aerobic exercise are unclear but might relate to upper versus lower body exercise and stimulation of muscle mass. Further investigation is warranted in this area given recent work suggesting cardiac adaptations to aerobic and resistance exercise are unique [42]. In either case, our findings extend upon this exercise work by showing favorable effects in the post-prandial period in older adults with prediabetes. 

Post-prandial gut hormones have been purported to influence vascular function [43,44,45]. Contrary to our hypothesis, gut hormones alone did not associate with any aortic waveforms measured in this study. This is surprising, given AG and dAG have both been implicated in vasomotor tone and nitric oxide-mediated endothelial function [43]. Indeed, AG and dAG were also both shown to inhibit ET-1-mediated vasoconstriction when applied to artery segments [43]. Additionally, GLP-1 has been shown to enhance muscle microvascular perfusion in healthy humans, as well as increase brachial artery diameter and flow velocity through PKA (protein kinase A)-mediated eNOS activation [45]. Lastly, GIP has been demonstrated to increase blood flow and triglyceride clearance in abdominal adipose tissue of lean humans via the recruitment of capillaries promoting lipoprotein lipase activity on triacylglycerol particles [43]. While much of these data demonstrate favorable effects of gut hormones on the vasculature, direct infusion, rather than oral ingestion, was used. Hence, we looked to expand on this work by testing if exercise would influence aortic waveforms via modulation of gut hormones during an OGTT. In our study, neither fasting or post-prandial AG, dAG, or GLP-1_active_ was altered compared with pre-intervention, and the change in these hormones did not correlate independently with aortic waveform responses. Why these hormones did not change more robustly is difficult to address, but total ghrelin often increases following weight loss greater than 3 kg, which is considerably more than the present study [46]. Another possibility is that gut hormone sensitivity changes may have occurred after exercise training, such that changes in gut hormones were not observed [47]. Indeed, recent work highlights improved GLP-1 sensitivity following endurance exercise in women with obesity. Given we did not measure gut hormone sensitivity, we cannot determine if a lack of change in hormones reflects a more sensitive system [48]. Regardless, our work suggests that two weeks of exercise is capable of improving aortic waveforms and blood pressure in older adults with prediabetes, and the gut hormones measured herein do not appear independently related.

In human endothelial cells, insulin binds to the insulin receptor (IR) and tyrosine kinase phosphorylates IRS-1. This phosphorylation leads to the downstream binding and activation of PI3K and Akt. Thereafter, Akt phosphorylates and activates eNOS for nitric oxide production [49]. Nitric oxide promotes the relaxation of smooth muscle cells lining the vessel walls, which ultimately increases perfusion and delivery of glucose and insulin to target tissues [37]. Endogenous insulin is influenced significantly by each of the gut hormones measured in this study. For example, a primary function of GLP-1 and GIP is the promotion of pancreatic insulin secretion [50]. Additionally, ghrelin has been reported to blunt beta-cell insulin secretion [51,52]. Thus, it would be reasonable to expect that changes in ambient insulin concentration during the post-prandial state might influence the relationship between gut hormones and aortic waveforms. Interestingly, dAG, AG, and GLP-1_active_ correlated with changes in aortic waveforms only after covarying for changes in insulin tAUC. Specifically, reductions in fasting dAG and AG tAUC were both associated with lowered Pb_120min_. This is clinically relevant as lower Pb suggests reduced impedances from the vascular walls producing a partial wave reflection back towards the heart [4,5]. Given, though, that ghrelin infusion has been shown to promote endothelial function, it is interesting that reductions in these hormones were associated with favorable Pb_120min_ results. A possible reason for this relates to ghrelin inducing reduced beta-cell function and/or promoting insulin resistance [53]. In the present study, insulin levels were reduced after both CONT and INT, perhaps suggesting the vasculature became more insulin responsive with less ambient ghrelin in circulation. Furthermore, this may explain the lack of change in post-prandial AG and dAG seen in both CONT and INT, as insulin infusion has been shown to decrease circulating total ghrelin [54]. Alternatively, it remains possible that the interaction between ghrelin and insulin influenced cellular signals (e.g., Akt) to modulate vessel function [55]. Indeed, lower fasting insulin correlated with lower Pf_120min_. This would be consistent with the reduced left ventricular workload and higher PPA seen with our intervention. In fact, increases in PPA are favorable as they demonstrate central arterial compliance leading to lower cPP relative to bPP [56]. Interestingly, increases in GLP-1_active_ tAUC_60min_ were associated with reduced Pf_180min_. Consistent with prior work, infusion of GLP-1 into healthy participants improved endothelial function [46]. In turn, better peripheral blood flow may enable greater delivery of insulin to reduce load on the left ventricle [57], which mirrors our reduced post-prandial AIx and AP, independent of heart rate, following exercise. Collectively, insulin appears to be a central post-prandial hormone regulating vascular function following exercise training in older adults with prediabetes.

This study has limitations that may impact our interpretations. The present study may be underpowered to detect statistical differences in some vascular outcomes. Based on tAUC data for Pf and Pb, the sample size required to detect an effect with 0.80 power at 0.05 significance was calculated for Pf (delta = 254, standard deviation (SD) = 687, *n* = 59) and Pb (delta = 117, SD = 446, *n* = 59) to inform future studies examining exercise and pressure waveforms. Interestingly, other work from our lab in women with obesity has similarly reported reductions in AIx75 following 2 weeks of exercise without concurrent changes in Pb or Pf [24]. Why we detect changes in AIx75, as well as blood pressure, is beyond the scope of this study but may be attributable to the software’s sensitivity to detect changes in the indirect measure of the waveforms during analysis and/or the use of an OGTT vs. direct hormone infusion, given insulin infusion has previously been shown to lower Pb in about 20 subjects [58]. A 75 g OGTT, rather than a mixed meal, was used to characterize post-prandial gut hormones. This may limit generalizability of the findings as macronutrients have been demonstrated to affect post-prandial ghrelin. For instance, ghrelin suppression occurs more from protein than carbohydrate or lipid-based meals [59]. Furthermore, food intake sequence has been shown to influence incretin responses. Incretin responses to carbohydrates in a meal are blunted when protein is consumed beforehand [60]. In either case, OGTT and mixed-meal tolerance tests show similar directional post-prandial gut hormone responses [61,62], with some alterations in magnitude of GLP-1 stimulation [63] and ghrelin suppression [64]. Another consideration is that gut hormones were measured at 0, 30, and 60 min of a 75 g OGTT. These limited timepoints may underestimate the effects of exercise on the hormones of interest. However, studies have demonstrated peak suppression of ghrelin, in addition to stimulation of GIP and GLP-1, occurs within the first 60 min of the OGTT [65], suggesting we are likely to depict initial gut hormone responses. However, it is possible that differences in gut hormone clearance rates could influence the vasculature. This study was also completed with an absence of healthy controls. Obesity status itself may blunt GLP-1 secretory responses to aerobic exercise [66], as well as mitigate AG increase following exercise [67]. Moreover, the present study features a modest sample size and primarily white women, highlighting additional attention to diverse groups of people is warranted. A non-exercise control was not included, so the independent effects of exercise may be over-/under-estimated. The study also consisted mostly of older white women, thereby limiting these findings to younger men and women from diverse backgrounds. Lastly, we used aortic waveforms to characterize vascular function. While some suggest AIx may be used as an indicator of arterial stiffness, it is worth nothing that pulse wave velocity (PWV) is considered the better non-invasive measure of arterial stiffness. Thus, we are not able to state whether gut hormones impact arterial stiffness, but instead focused on analysis of changes in aortic load and/or peripheral arterial compliance. 

In conclusion, two weeks of exercise improved post-prandial aortic waveforms in older adults with prediabetes, independent of intensity. Furthermore, CONT exercise favored reductions in post-prandial blood pressure when compared with INT exercise. While gut hormone changes after exercise training were not independently related to improvements in central hemodynamics, covarying for insulin revealed significant relationships. This observation suggests gut hormones may interact with insulin to influence aortic waveforms in older adults with prediabetes. Therefore, additional studies are necessary to elucidate the underlying pancreatic-gut “cross-talk” mechanism with the vasculature to optimize CVD risk reduction.

## Figures and Tables

**Table 1:** Effect of continuous and interval exercise on anthropometrics, fitness, and glycemia.

	CONT		INT		ANOVA *p* Value	
	Pre	Post	Pre	Post	Test	G × T
n (female)	14 (11)		14 (11)			
Non-Hispanic white	12		13			
Non-Hispanic black	1		1			
Hispanic	1		0			
Age, yr	62.1 ± 2.2		60.4 ± 2.0			
Weight, kg	94.3 ± 4.7	94.0 ± 4.7	88.7 ± 3.6	87.9 ± 3.7	<0.01	0.07
BMI, kg/m^2^	34.5 ± 1.9	34.4 ±1.9	32.1 ±1.3	31.8 ± 1.3	<0.01	0.12
VO_2_peak, mL⋅kg^−1^⋅min^−1^	20.1 ± 1.2	20.6 ± 1.5	20.3 ± 1.1	22.1 ± 1.1	0.01	0.13
Glucose						
Fasting, mg/dL	105.2 ± 2.5	103.3 ± 3.3	101.7 ± 1.7	102.7 ± 2.2	0.74	0.36
120 min, mg/dL	145.3 ± 8.7	132.9 ± 8.3	146.7 ± 11.1	128.9 ± 10.1	0.02	0.68
tAUC, mg/dL × 180 min	25,804.3 ± 1379.6	23,830.7 ± 1303.4	25,184.6 ± 1469.1	23,664.4 ± 1285.4	0.03	0.83

Note: Data are mean ± SEM. CONT = continuous exercise. INT = interval exercise. BMI = body mass index. VO_2_peak = peak oxygen consumption. tAUC = total area under the curve.

**Table 2:** Effect of continuous and interval exercise on fasting and post-prandial hormones.

	CONT		INT		ANOVA *p* Value	
	Pre	Post	Pre	Post	Test	G × T
AG						
Fasting, pg/mL	66.1 ± 12.5	65.1 ± 10.0	83.3 ± 15.9	66.4 ± 8.9	0.32	0.41
tAUC × 60 min, pg/mL	1229.1 ± 206.6	1130.3 ± 135.4	1192.2 ± 167.4	1005.6 ± 127.3	0.20	0.94
dAG						
Fasting, pg/mL	49.5 ± 8.4	52.5 ± 5.9	71.6 ± 13.5	64.8 ± 14.5	0.66	0.55
tAUC × 60 min, pg/mL	1049.5 ± 166.3	1186.3 ± 170.8	1615.0 ± 301.1	1481.0 ± 345.9	0.72	0.54
GIP						
Fasting, pg/mL	62.9 ± 11.2	73.0 ± 15.1	59.5 ± 5.3	46.0 ± 7.0	0.73	0.03
tAUC × 60 min, pg/mL	8419.4 ± 1222.1	8666.7 ± 1175.0	10340.3 ± 603.6	10010.6 ± 1006.8	0.93	0.41
GLP-1						
Fasting, pg/mL	5.9 ± 1.9	7.9 ± 2.3	6.3 ± 1.4	5.2 ± 1.1	0.38	0.12
tAUC × 60 min, pg/mL	337.6 ± 90.3	413.2 ± 97.0	378.8 ± 48.7	368.3 ± 44.8	0.73	0.26
Insulin						
Fasting, μU/mL	13.1 ± 2.5	12.3 ± 2.1	12.2 ± 2.3	12.1 ± 2.5	0.65	0.57
120 min, μU/mL	101.0 ± 16.8	80.1 ± 11.6	93.0 ± 18.5	76.6 ± 15.9	<0.01	0.95
tAUC × 180 min, μU/mL	14,232.1 ± 2143.5	12,051.5 ± 1829.2	14,482.1 ± 2027.2	12,330.4 ± 1770.1	<0.01	0.98

Note: Data are mean ± SEM. CONT = continuous exercise. INT = interval exercise. AG = acyl ghrelin. dAG = des-acyl ghrelin. GIP = glucose-dependent insulinotropic polypeptide. GLP-1 = glucagon-like peptide 1.

**Table 3:** Effects of continuous and interval exercise on central and peripheral hemodynamics.

	CONT		INT		ANOVA *p* Value	
	Pre	Post	Pre	Post	Test	G × T
bSBP						
Fasting, mmHg	131.1 ± 3.2	133.9 ± 3.4	140.2 ± 3.7	143.3 ± 5.0	0.66	0.58
120 min, mmHg	135.9 ± 4.2	135.7 ± 3.8	143.4 ± 5.1	145.0 ± 4.6	0.57	0.88
tAUC, mmHg × 180 min	24,681.4 ± 614.5	23,196.4 ± 844.6	25,412.3 ± 620.3	25,772.1 ± 654.3	0.34	0.02
cSBP						
Fasting, mmHg	124.9 ± 3.3	124.9 ± 3.1	134.9 ± 3.9	132.9 ± 4.9	0.14	0.09
120 min, mmHg	125.3 ± 3.6	123.0 ± 3.4	131.5 ± 5.0	130.4 ± 4.6	0.96	0.82
tAUC, mmHg × 180 min	22,866.9 ± 546.6	22,107.5 ± 479.5	23,225.0 ± 562.0	23,185.4 ± 620.3	0.45	0.32
bDBP						
Fasting, mmHg	79.6 ± 3.1	81.1 ± 2.9	85.3 ± 3.5	83.7 ± 3.0	0.92	0.53
120 min, mmHg	79.0 ± 2.3	78.4 ± 2.4	80.0 ± 3.1	81.6 ± 2.9	0.45	0.19
tAUC, mmHg × 180 min	14,145.0 ± 392.9	13,305.0 ± 60.1	14,261.5 ± 351.0	14,481.4 ± 394.7	0.46	0.04
cDBP						
Fasting, mmHg	80.9 ± 3.1	82.4 ± 2.9	84.5 ± 3.2	85.1 ± 3.0	0.98	0.55
120 min, mmHg	80.1 ± 2.2	74.4 ± 2.4	81.1 ± 3.0	83.6 ± 2.9	0.33	0.19
tAUC, mmHg × 180 min	14,449.3 ± 394.8	14,287.5 ± 414.0	14,513.1 ± 343.5	14,755.7 ± 402.4	0.89	0.41
bPP						
Fasting, mmHg	54.3 ± 2.7	52.8 ± 2.9	62.4 ± 2.5	59.6 ± 3.6	0.23	0.80
120 min, mmHg	56.9 ± 3.4	57.3 ± 3.2	63.4 ± 4.1	63.4 ± 3.2	0.98	0.99
tAUC, mmHg × 180 min	10,620.0 ± 467.1	10,210.0 ± 401.3	11,173.8 ± 565.9	11,290.7 ± 483.7	0.58	0.10
cPP						
Fasting, mmHg	44.0 ± 2.4	42.6 ± 2.5	50.6 ± 2.2	47.8 ± 3.4	0.16	0.98
120 min, mmHg	44.1 ± 2.8	43.2 ± 2.5	49.6 ± 3.5	47.3 ± 2.9	0.75	0.37
tAUC, mmHg × 180 min	8247.7 ± 423.8	7820.0 ± 321.6	8651.5 ± 473.6	8520.0 ± 414.7	0.35	0.34
AIx						
Fasting, %	28.4 ± 2.3	28.8 ± 2.5	28.5 ± 3.3	25.9 ± 3.0	0.56	0.55
120 min, %	24.7 ± 2.1	18.4 ± 1.9	23.6 ± 3.2	17.0 ± 2.9	<0.01	0.66
tAUC, % × 180 min	4753.8 ± 278.1	3730.7 ± 280.3	4097.1 ± 386.5	3398.6 ± 419.5	<0.01	0.72
AP						
Fasting, mmHg	15.4 ± 1.1	15.6 ± 1.4	19.1 ± 2.2	16.7 ± 2.2	0.26	0.35
120 min, mmHg	13.6 ± 1.3	10.8 ± 1.1	16.5 ± 1.8	10.6 ± 2.1	0.02	0.52
tAUC, mmHg × 180 min	2809.3 ± 209.8	2222.5 ± 162.8	2765.0 ± 242.0	2190.0 ± 256.5	<0.01	0.79
HR						
Fasting, beats min^−1^	60.0 ± 2.4	60.0 ± 2.9	58.7 ± 3.0	59.8 ± 2.2	0.66	0.22
120 min, beats min^−1^	62.9 ± 1.9	61.9 ± 2.2	61.1 ± 2.4	62.2 ± 1.9	0.94	0.35
tAUC, mmHg × 180 min	11,259.2 ± 348.0	11,357.5 ± 432.5	11,113.8 ± 429	11,112.5 ± 325.2	0.73	0.85

Note: Data are mean ± SEM. CONT = continuous exercise. INT = interval exercise. bSBP = brachial systolic blood pressure. cSBP = central systolic blood pressure. bDBP = brachial diastolic blood pressure. cDBP = central diastolic blood pressure, bPP = brachial pulse pressure, cPP = central pulse pressure, AIx = augmentation index, AP = augmentation pressure, and HR = heart rate.

## Data Availability

These data have not been made publicly available since no repository is currently available. However, the corresponding author (SKM) can provide further information on the data upon reasonable request.

## References

[1] Palatini P., Casiglia E., Gąsowski J., Głuszek J., Jankowski P., Narkiewicz K., Saladini F., Stolarz-Skrzypek K., Tikhonoff V., Bortel L.V. (2011). Arterial Stiffness, Central Hemodynamics, and Cardiovascular Risk in Hypertension. Vasc. Health Risk Manag..

[2] Chester R.C., Gornbein J.A., Hundley W.G., Srikanthan P., Watson K.E., Horwich T. (2017). Reflection Magnitude, a Measure of Arterial Stiffness, Predicts Incident Heart Failure in Men But Not Women: Multi-Ethnic Study of Atherosclerosis (MESA). J. Card. Fail..

[3] Mitchell G.F., Hwang S.-J., Vasan R.S., Larson M.G., Pencina M.J., Hamburg N.M., Vita J.A., Levy D., Benjamin E.J. (2010). Arterial Stiffness and Cardiovascular Events: The Framingham Heart Study. Circulation.

[4] Townsend R.R., Wilkinson I.B., Schiffrin E.L., Avolio A.P., Chirinos J.A., Cockcroft J.R., Heffernan K.S., Lakatta E.G., McEniery C., Mitchell G.F. (2015). Recommendations for Improving and Standardizing Vascular Research on Arterial Stiffness. Hypertension.

[5] Stock J.M., Chouramanis N.V., Chirinos J.A., Edwards D.G. (2020). Dynamic and Isometric Handgrip Exercise Increases Wave Reflection in Healthy Young Adults. J. Appl. Physiol. (1985).

[6] Laurent S. (2006). Surrogate Measures of Arterial Stiffness. Hypertension.

[7] Widmer R.J., Lerman A. (2014). Endothelial Dysfunction and Cardiovascular Disease. Glob. Cardiol. Sci. Pract..

[8] Cavalot F., Petrelli A., Traversa M., Bonomo K., Fiora E., Conti M., Anfossi G., Costa G., Trovati M. (2006). Post-prandial Blood Glucose Is a Stronger Predictor of Cardiovascular Events than Fasting Blood Glucose in Type 2 Diabetes Mellitus, Particularly in Women: Lessons from the San Luigi Gonzaga Diabetes Study. J. Clin. Endocrinol. Metab..

[9] Shirwany N.A., Zou M. (2010). Arterial Stiffness: A Brief Review. Acta Pharmacol. Sin..

[10] Papaioannou T.G., Protogerou A.D., Stergiopulos N., Vardoulis O., Stefanadis C., Safar M., Blacher J. (2014). Total Arterial Compliance Estimated by a Novel Method and All-Cause Mortality in the Elderly: The PROTEGER Study. Age (Dordr.).

[11] Westerbacka J., Yki-Järvinen H. (2002). Arterial Stiffness and Insulin Resistance. Semin. Vasc. Med..

[12] Jahn L.A., Hartline L., Rao N., Logan B., Kim J.J., Aylor K., Gan L.-M., Westergren H.U., Barrett E.J. (2016). Insulin Enhances Endothelial Function Throughout the Arterial Tree in Healthy But Not Metabolic Syndrome Subjects. J. Clin. Endocrinol. Metab..

[13] Westerbacka J., Vehkavaara S., Bergholm R., Wilkinson I., Cockcroft J., Yki-Järvinen H. (1999). Marked Resistance of the Ability of Insulin to Decrease Arterial Stiffness Characterizes Human Obesity. Diabetes.

[14] Jatic Z., Skopljak A., Hebibovic S., Sukalo A., Rustempasic E., Valjevac A. (2019). Effects of Different Antihypertensive Drug Combinations on Blood Pressure and Arterial Stiffness. Med. Arch..

[15] Dotson B.L., Heiston E.M., Miller S.L., Malin S.K. (2021). Insulin Stimulation Reduces Aortic Wave Reflection in Adults with Metabolic Syndrome. Am. J. Physiol. Heart Circ. Physiol..

[16] Nagaya N., Kojima M., Uematsu M., Yamagishi M., Hosoda H., Oya H., Hayashi Y., Kangawa K. (2001). Hemodynamic and Hormonal Effects of Human Ghrelin in Healthy Volunteers. Am. J. Physiol.-Regul. Integr. Comp. Physiol..

[17] Nagaya N., Moriya J., Yasumura Y., Uematsu M., Ono F., Shimizu W., Ueno K., Kitakaze M., Miyatake K., Kangawa K. (2004). Effects of Ghrelin Administration on Left Ventricular Function, Exercise Capacity, and Muscle Wasting in Patients with Chronic Heart Failure. Circulation.

[18] Dixit V.D., Schaffer E.M., Pyle R.S., Collins G.D., Sakthivel S.K., Palaniappan R., Lillard J.W., Taub D.D. (2004). Ghrelin Inhibits Leptin- and Activation-Induced Proinflammatory Cytokine Expression by Human Monocytes and T Cells. J. Clin. Investig..

[19] Tesauro M., Schinzari F., Iantorno M., Rizza S., Melina D., Lauro D., Cardillo C. (2005). Ghrelin Improves Endothelial Function in Patients with Metabolic Syndrome. Circulation.

[20] Tan A.W.K., Subaran S.C., Sauder M.A., Chai W., Jahn L.A., Fowler D.E., Patrie J.T., Aylor K.W., Basu A., Liu Z. (2018). GLP-1 and Insulin Recruit Muscle Microvasculature and Dilate Conduit Artery Individually But Not Additively in Healthy Humans. J. Endocr. Soc..

[21] Asmar M., Asmar A., Simonsen L., Dela F., Holst J.J., Bülow J. (2019). GIP-Induced Vasodilation in Human Adipose Tissue Involves Capillary Recruitment. Endocr. Connect..

[22] Asmar M., Simonsen L., Madsbad S., Stallknecht B., Holst J.J., Bülow J. (2010). Glucose-Dependent Insulinotropic Polypeptide May Enhance Fatty Acid Re-Esterification in Subcutaneous Abdominal Adipose Tissue in Lean Humans. Diabetes.

[23] Asmar M., Simonsen L., Arngrim N., Holst J.J., Dela F., Bülow J. (2014). Glucose-Dependent Insulinotropic Polypeptide Has Impaired Effect on Abdominal, Subcutaneous Adipose Tissue Metabolism in Obese Subjects. Int. J. Obes. (Lond.).

[24] Heiston E.M., Gilbertson N.M., Eichner N.Z.M., Malin S.K. (2021). A Low-Calorie Diet with or without Exercise Reduces Post-prandial Aortic Waveform in Females with Obesity. Med. Sci. Sports Exerc..

[25] Gilbertson N.M., Eichner N.Z.M., Heiston E.M., Gaitán J.M., Francois M.E., Mehaffey J.H., Hassinger T.E., Hallowell P.T., Weltman A., Malin S.K. (2019). A Low-Calorie Diet with or without Interval Exercise Training Improves Adiposopathy in Obese Women. Appl. Physiol. Nutr. Metab..

[26] Gaesser G.A., Angadi S.S., Sawyer B.J. (2011). Exercise and Diet, Independent of Weight Loss, Improve Cardiometabolic Risk Profile in Overweight and Obese Individuals. Phys. Sportsmed..

[27] Stock J.M., Chirinos J.A., Edwards D.G. (2021). Lower-Body Dynamic Exercise Reduces Wave Reflection in Healthy Young Adults. Exp. Physiol..

[28] Hanssen H., Nussbaumer M., Moor C., Cordes M., Schindler C., Schmidt-Trucksäss A. (2015). Acute Effects of Interval versus Continuous Endurance Training on Pulse Wave Reflection in Healthy Young Men. Atherosclerosis.

[29] Munir S., Jiang B., Guilcher A., Brett S., Redwood S., Marber M., Chowienczyk P. (2008). Exercise Reduces Arterial Pressure Augmentation through Vasodilation of Muscular Arteries in Humans. Am. J. Physiol.-Heart Circ. Physiol..

[30] Goto C., Nishioka K., Umemura T., Jitsuiki D., Sakagutchi A., Kawamura M., Chayama K., Yoshizumi M., Higashi Y. (2007). Acute Moderate-Intensity Exercise Induces Vasodilation through an Increase in Nitric Oxide Bioavailiability in Humans*. Am. J. Hypertens..

[31] Eichner N.Z.M., Gaitán J.M., Gilbertson N.M., Khurshid M., Weltman A., Malin S.K. (2019). Post-prandial Augmentation Index Is Reduced in Adults with Prediabetes Following Continuous and Interval Exercise Training. Exp. Physiol..

[32] Benetos A., Thomas F., Joly L., Blacher J., Pannier B., Labat C., Salvi P., Smulyan H., Safar M.E. (2010). Pulse Pressure Amplification a Mechanical Biomarker of Cardiovascular Risk. J. Am. Coll. Cardiol..

[33] Heiston E.M., Eichner N.Z.M., Gilbertson N.M., Gaitán J.M., Kranz S., Weltman A., Malin S.K. (2019). Two Weeks of Exercise Training Intensity on Appetite Regulation in Obese Adults with Prediabetes. J. Appl. Physiol. (1985).

[34] Malin S.K., Francois M.E., Eichner N.Z.M., Gilbertson N.M., Heiston E.M., Fabris C., Breton M. (2018). Impact of Short-Term Exercise Training Intensity on β-Cell Function in Older Obese Adults with Prediabetes. J. Appl. Physiol. (1985).

[35] John A.T., Chowdhury M., Islam M.R., Mir I.A., Hasan M.Z., Chong C.Y., Humayra S., Higashi Y. (2022). Effectiveness of High-Intensity Interval Training and Continuous Moderate-Intensity Training on Blood Pressure in Physically Inactive Pre-Hypertensive Young Adults. J. Cardiovasc. Dev. Dis..

[36] Malin S.K., Gilbertson N.M., Eichner N.Z.M., Heiston E., Miller S., Weltman A. (2019). Impact of Short-Term Continuous and Interval Exercise Training on Endothelial Function and Glucose Metabolism in Prediabetes. J. Diabetes Res..

[37] Mikus C.R., Fairfax S.T., Libla J.L., Boyle L.J., Vianna L.C., Oberlin D.J., Uptergrove G.M., Deo S.H., Kim A., Kanaley J.A. (2011). Seven Days of Aerobic Exercise Training Improves Conduit Artery Blood Flow Following Glucose Ingestion in Patients with Type 2 Diabetes. J. Appl. Physiol..

[38] Shenouda N., Gillen J.B., Gibala M.J., MacDonald M.J. (2017). Changes in Brachial Artery Endothelial Function and Resting Diameter with Moderate-Intensity Continuous but Not Sprint Interval Training in Sedentary Men. J. Appl. Physiol..

[39] Tai Y.L., Gerhart H., Mayo X., Kingsley J.D. (2018). Acute Resistance Exercise Using Free Weights on Aortic Wave Reflection Characteristics. Clin. Physiol. Funct. Imaging.

[40] Marshall E.M., Parks J.C., Singer T.J., Tai Y.L., DeBord A.R., Humm S.M., Kingsley J.D. (2021). Vascular Responses to High-Intensity Battling Rope Exercise between the Sexes. J. Sports Sci. Med..

[41] Patik J.C., Stock J.M., Shenouda N., Chouramanis N.V., Mehrer J.D., Chirinos J.A., Edwards D.G. (2021). Pulsatile Load and Wasted Pressure Effort Are Reduced Following an Acute Bout of Aerobic Exercise. J. Appl. Physiol. (1985).

[42] Marsh C.E., Thomas H.J., Naylor L.H., Dembo L.G., Scurrah K.J., Green D.J. (2022). Left Ventricular Adaptation to Exercise Training via Magnetic Resonance Imaging: Studies of Twin Responses to Understand Exercise THerapy. Med. Sci. Sports Exerc..

[43] Pearson J.T., Shirai M., Sukumaran V., Du C.-K., Tsuchimochi H., Sonobe T., Waddingham M.T., Katare R., Schwenke D.O. (2019). Ghrelin and Vascular Protection. Vasc. Biol..

[44] Broom D.R., Stensel D.J., Bishop N.C., Burns S.F., Miyashita M. (2007). Exercise-Induced Suppression of Acylated Ghrelin in Humans. J. Appl. Physiol..

[45] Subaran S.C., Sauder M.A., Chai W., Jahn L.A., Fowler D.E., Aylor K.W., Basu A., Liu Z. (2014). GLP-1 at Physiological Concentrations Recruits Skeletal and Cardiac Muscle Microvasculature in Healthy Humans. Clin. Sci..

[46] Leidy H.J., Gardner J.K., Frye B.R., Snook M.L., Schuchert M.K., Richard E.L., Williams N.I. (2004). Circulating Ghrelin Is Sensitive to Changes in Body Weight during a Diet and Exercise Program in Normal-Weight Young Women. J. Clin. Endocrinol. Metab..

[47] Mani B.K., Castorena C.M., Osborne-Lawrence S., Vijayaraghavan P., Metzger N.P., Elmquist J.K., Zigman J.M. (2018). Ghrelin Mediates Exercise Endurance and the Feeding Response Post-Exercise. Mol. Metab..

[48] Åkerström T., Stolpe M.N., Widmer R., Dejgaard T.F., Højberg J.M., Møller K., Hansen J.S., Trinh B., Holst J.J., Thomsen C. (2022). Endurance Training Improves GLP-1 Sensitivity and Glucose Tolerance in Overweight Women. J. Endocr. Soc..

[49] Muniyappa R., Montagnani M., Koh K.K., Quon M.J. (2007). Cardiovascular Actions of Insulin. Endocr. Rev..

[50] Vilsbøll T., Holst J.J. (2004). Incretins, Insulin Secretion and Type 2 Diabetes Mellitus. Diabetologia.

[51] McLaughlin T., Abbasi F., Lamendola C., Frayo R.S., Cummings D.E. (2004). Plasma Ghrelin Concentrations Are Decreased in Insulin-Resistant Obese Adults Relative to Equally Obese Insulin-Sensitive Controls. J. Clin. Endocrinol. Metab..

[52] Broglio F., Arvat E., Benso A., Gottero C., Muccioli G., Papotti M., van der Lely A.J., Deghenghi R., Ghigo E. (2001). Ghrelin, a Natural GH Secretagogue Produced by the Stomach, Induces Hyperglycemia and Reduces Insulin Secretion in Humans. J. Clin. Endocrinol. Metab..

[53] Vestergaard E.T., Gormsen L.C., Jessen N., Lund S., Hansen T.K., Moller N., Jorgensen J.O.L. (2008). Ghrelin Infusion in Humans Induces Acute Insulin Resistance and Lipolysis Independent of Growth Hormone Signaling. Diabetes.

[54] McCowen K.C., Maykel J.A., Bistrian B.R., Ling P.R. (2002). Circulating Ghrelin Concentrations Are Lowered by Intravenous Glucose or Hyperinsulinemic Euglycemic Conditions in Rodents. J. Endocrinol..

[55] Baldanzi G., Filigheddu N., Cutrupi S., Catapano F., Bonissoni S., Fubini A., Malan D., Baj G., Granata R., Broglio F. (2002). Ghrelin and Des-Acyl Ghrelin Inhibit Cell Death in Cardiomyocytes and Endothelial Cells through ERK1/2 and PI 3-Kinase/AKT. J. Cell Biol..

[56] Nijdam M.-E., Plantinga Y., Hulsen H.T., Bos W.J.W., Grobbee D.E., van der Schouw Y.T., Bots M.L. (2008). Pulse Pressure Amplification and Risk of Cardiovascular Disease. Am. J. Hypertens..

[57] Love K.M., Liu J., Regensteiner J.G., Reusch J.E.B., Liu Z. (2020). GLP-1 and Insulin Regulation of Skeletal and Cardiac Muscle Microvascular Perfusion in Type 2 Diabetes. J. Diabetes.

[58] Remchak M.-M.E., Heiston E.M., Ballantyne A., Dotson B.L., Malin S.K. (2022). Aortic. Waveform Responses to Insulin in Late versus Early Chronotype with Metabolic Syndrome. Physiol. Rep..

[59] Foster-Schubert K.E., Overduin J., Prudom C.E., Liu J., Callahan H.S., Gaylinn B.D., Thorner M.O., Cummings D.E. (2008). Acyl and Total Ghrelin Are Suppressed Strongly by Ingested Proteins, Weakly by Lipids, and Biphasically by Carbohydrates. J. Clin. Endocrinol. Metab..

[60] Sun L., Goh H.J., Govindharajulu P., Leow M.K.-S., Henry C.J. (2020). Post-prandial Glucose, Insulin and Incretin Responses Differ by Test Meal Macronutrient Ingestion Sequence (PATTERN Study). Clin. Nutr..

[61] Brown M.A., Green B.P., James L.J., Stevenson E.J., Rumbold P.L.S. (2016). The Effect of a Dairy-Based Recovery Beverage on Post-Exercise Appetite and Energy Intake in Active Females. Nutrients.

[62] Yau A.M.W., McLaughlin J., Gilmore W., Maughan R.J., Evans G.H. (2017). The Acute Effects of Simple Sugar Ingestion on Appetite, Gut-Derived Hormone Response, and Metabolic Markers in Men. Nutrients.

[63] Koopman A.D.M., Rutters F., Rauh S.P., Nijpels G., Holst J.J., Beulens J.W., Alssema M., Dekker J.M. (2018). Incretin Responses to Oral Glucose and Mixed Meal Tests and Changes in Fasting Glucose Levels during 7 Years of Follow-up: The Hoorn Meal Study. PLoS ONE.

[64] Codella R., Benedini S., Paini S., Caumo A., Adamo M., Terruzzi I., Ferrulli A., Macrì C., Andreoni L., Sterlicchio M. (2017). Effect of Sugar versus Mixed Breakfast on Metabolic and Neurofunctional Responses in Healthy Individuals. J. Diabetes Res..

[65] Gjesing A.P., Ekstrøm C.T., Eiberg H., Urhammer S.A., Holst J.J., Pedersen O., Hansen T. (2012). Fasting and Oral Glucose-Stimulated Levels of Glucose-Dependent Insulinotropic Polypeptide (GIP) and Glucagon-like Peptide-1 (GLP-1) Are Highly Familial Traits. Diabetologia.

[66] Adam T.C.M., Westerterp-Plantenga M.S. (2004). Activity-Induced GLP-1 Release in Lean and Obese Subjects. Physiol. Behav..

[67] Mackelvie K.J., Meneilly G.S., Elahi D., Wong A.C.K., Barr S.I., Chanoine J.-P. (2007). Regulation of Appetite in Lean and Obese Adolescents after Exercise: Role of Acylated and Desacyl Ghrelin. J. Clin. Endocrinol. Metab..

